# Effect of ultrasonication and use of aminopeptidase from *Aeromonas proteolytica* on quality parameters of fish protein hydrolysates from side streams of Atlantic mackerel (*Scomber scombrus*)

**DOI:** 10.3389/fnut.2025.1591672

**Published:** 2025-06-13

**Authors:** Janna Cropotova, Kristine Kvangarsnes, Janne Stangeland, Amélie Le Gall, Turid Rustad

**Affiliations:** ^1^Department of Biological Sciences Ålesund, Norwegian University of Science and Technology, Ålesund, Norway; ^2^Møreforsking AS, Ålesund, Norway; ^3^L’institut Agro Dijon, Dijon, France; ^4^Department of Biotechnology and Food Science, Norwegian University of Science and Technology, Trondheim, Norway

**Keywords:** ultrasound treatment, enzymatic hydrolysis, FPH, Atlantic mackerel, aminopeptidase

## Abstract

In the present study, both ultrasonication (US) and enzymatic treatment with aminopeptidase from *Aeromonas proteolytica* (AAP) were applied as a post-treatment to fish protein hydrolysates (FPH) recovered from Atlantic mackerel. The single and combined effects of AAP and US treatments at 300 W and 500 W at 20 kHz for 10 min on the physicochemical characteristics of FPH were assessed. The results showed a significant decrease (*p* < 0.05) in soluble proteins after application of US treatment of FPH at 300 W and 500 W (79.1 and 70.3%, respectively) and US treatment at 300 W and 500 W together with AAP (50.3 and 72.5%, respectively) compared to control (84.4%). This is due to cavitation effect of sonication resulting in aggregation of peptides. This decrease was accompanied by a significant decrease in thiol groups in all experimental FPH samples varying from 5.85 to 9.54 nmol/mg compared to control (12.86 nmol/mg). At the same time, there was a significant increase in the distribution of small peptides with molecular weight (MW) between 200 and 1,000 Da along with a significant decrease in medium-size peptides with MW of 1,000 and 5,000 Da in all AAP-treated FPH samples compared to FPH without AAP. The proportion of essential amino acids increased significantly (*p* < 0.05) in all experimental FPH samples varying from 28.2 to 29.1% except for 500 W + AAP (24.2%) compared to control (25.7%), revealing a positive combined effect of AAP and US treatment on nutritional profile of mackerel hydrolysates. However, there was no significant difference in the proportion of hydrophobic free amino acids responsible for bitter taste between any of experimental FPH samples and control. Regarding color parameters, there was a significant increase in lightness accompanied by a significant decrease in redness in all US-treated FPH samples compared to control due to ultrasound-induced cavitation effect changing the secondary structure of peptide molecules. The novel approach of combined use of US and AAP to improving physicochemical parameters of mackerel FPH may provide valuable insights into process optimization for enhanced quality and functional properties of fish protein ingredients.

## Graphical Abstract

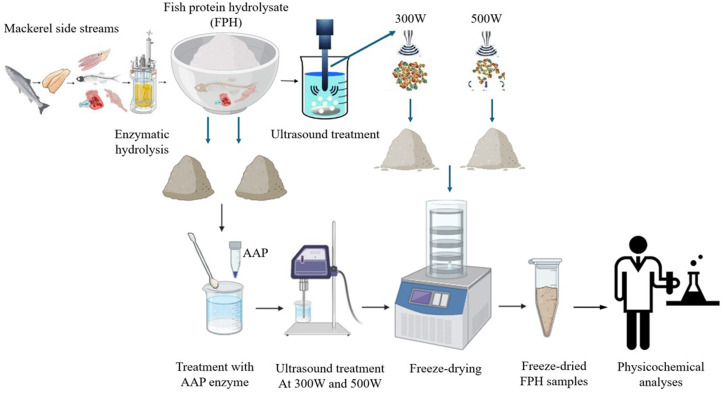

## 1 Introduction

The global population is continuously increasing and is projected to reach 9.7 billion by 2050 (1). As a result, the demand for high-quality protein ingredients containing all amino acids is rising as more and more people are consuming proteins to cover their nutritional needs (2). However, currently approximately 60% of global protein production is used to feed cattle, animals, and farmed fish (3). Therefore, there is a strong need to find new sources of high-quality protein ingredients to meet the demand of the growing population. Fish is one of the best sources of complete protein, providing all essential amino acids in adequate proportions necessary in the human diet, with a digestibility rate of approximately 98.3–98.8% (4). The high digestibility is mainly due to the low content of connective tissue proteins, i.e., collagen and elastin in the fish muscle (5). The total protein content in the muscles of the most fish species varies from 12.2 to 21.8%, while the amounts of essential amino acids fulfill the amino acid scoring profile for adults (4). However, only 30–40% of the fish production is intended for direct human consumption as fish steaks, fillets, medallions, etc. (6). Side streams (skin, heads, viscera, bones, and backbones) make up the remaining 60–70% of the fish after processing. These side streams are normally discarded or used for low value applications such as fish meal or oil (6). However, they may be used for recovery of valuable protein compounds with functional and health-promoting properties (7).

One of the ways to obtain high-value protein compounds with increased digestibility, bioavailability, and bioactive properties is to hydrolyze seafood rest raw material (8). Several methods are used to hydrolyze fish side streams to produce fish protein hydrolysates (FPH), including chemical hydrolysis, autolysis, and enzymatic hydrolysis (9). Due to a number of benefits, enzymatic hydrolysis is one of the most commonly used methods to produce FPH for human consumption and pet food (10). The main technological advantages are shorter reaction time, opportunity to control the process to produce specific hydrolysates, retention of the nutritive value of the primary protein, and lack of residual organic solvents (10). Enzymatic hydrolysis is a process that cleaves proteins into a mixture of peptides of different sizes and free amino acids (FAAs), producing protein hydrolysates (9). During this process, the size of peptides is reduced, while the number of carboxyl and amino groups is increased, resulting in changes in the protein structure and improved functional properties (solubility, emulsifying, and foaming) and bioavailability (11). In addition, FPH possess many biological activities and health-promoting effects such as antimicrobial, antioxidative, antihypertensive, immunomodulatory, and anti-inflammatory activity depending on the molecular size of the peptides (12). Thus, FPH represents a valuable ingredient, which when obtained from underutilized fish side streams would bring added value to the fish processing industry as one of the most sustainable and environmentally friendly ways to utilize the generated fish residual material. However, the functionality of FPH is strongly affected by the choice of enzymes and the degree to which the protein is hydrolyzed, as well as by the reaction conditions during enzymatic hydrolysis (9). The production of FPH involves enzymes such as endopeptidases and exopeptidases participating in the proteolysis process.

There are several efficient proteolytic enzymes including pepsin, trypsin, alcalase, neutrase, papain, and bromelain which are commonly used to obtain FPH (9). Alcalase is an endopeptidase which breaks down peptide bonds from C-terminal amino acids. Alcalase has been found to be a highly efficient enzyme for the production of FPH with small-sized peptides in a relatively short time (13). However, endopeptidases with broad specificity such as alcalase have been shown to result in higher bitterness of FPH through the generation of higher amounts of hydrophobic free amino acids and small peptides (13). Normally, the bitterness of peptides depends on the free amino acid composition, proportion of small peptides, and sequence of amino acids in the peptides, as well as hydrophobic properties of free amino acids (14). Indeed, small peptides and free amino acids with an average residue hydrophobicity greater than 1.4 kcal/mol are considered bitter. These include cysteine, isoleucine, leucine, methionine, phenylalanine, tryptophan, and valine (15). On the contrary, exopeptidases, such as, for example, aminopeptidases, are widely used in the food industry as a debittering agent for brewing, baking, and cheese making processes due to their capacity to remove the bitterness of peptides (16). These peptidases belong to a class of proteases that catalyze the cleavage of the amino terminal of amino acid residues in proteins or and peptide molecules (16). The debittering property of aminopeptidases has been largely demonstrated in various studies, including the reduction of bitterness in FPH (16–18). Studies have demonstrated that using aminopeptidase helps to reduce bitterness by releasing free amino acids such as phenylalanine, isoleucine, or leucine (16–18).

One of the group of aminopeptidases that hydrolyze a wide range of N-terminal amino acid residues from proteins and polypeptides is aminopeptidase from *Aeromonas proteolytica* (AAP). AAP has been shown to be one of the most stable enzymes. This aminopeptidase may retain its enzymatic activity at a temperature of 70°C for several hours and may only partially be inactivated in urea (16–18). These unique characteristics may be used to perform advanced enzymatic modification of peptides or protein hydrolysates coupled with innovative technological treatments such as ultrasonication, to improve functional (protein solubility, emulsification, etc.), nutritional (amino acid profile), and quality (bitterness, color, degree of hydrolysis, etc.) parameters of protein ingredients (19, 20).

In addition to a proper selection of enzymes, it is also important to apply efficient and safe extraction procedures to recover valuable compounds from fish side streams. Regardless of the fact that conventional enzymatic hydrolysis is a very effective method for protein and lipid extraction, its primary drawback relates to high costs of enzymes and risk of thermal degradation of both protein and lipid compounds due to high processing temperatures (12). Alternatively, replacing expensive enzymes by organic solvents such as formic acid to perform acid hydrolysis of fish side streams during silaging carries certain health and environmental risks and is limited to recovery of low value products such as fish meal and oil (21, 22). Recently, a number of non-thermal and environmentally friendly methods of extraction of valuable ingredients from food and seafood side streams to support green technology have been adopted in the food industry (12). Among these advanced extraction techniques are high pressure processing (HPP), ultrasound-assisted (US) extraction, supercritical fluid extraction (SFE), and microwave-assisted extraction (MAE) (23). These technologies have several advantages including no or minimal need in organic solvents, fast rate of extraction, improved compound recovery, enhanced selectivity (24), and are thus widely recognized as green and environmentally friendly methods for recovery of valuable compounds from fish side streams (6, 23). Among all these technologies, ultrasound-assisted hydrolysis has proven its effectiveness in obtaining higher recovery yields of FPH from trout by-products when compared to conventional enzymatic hydrolysis (25). Moreover, other studies have shown that US treatment can significantly improve quality parameters and health benefits of FPH extracted from Atlantic mackerel (12).

The present study investigated how the use of ultrasound treatment with and without application of exopeptidase influences the quality of FPH obtained by enzymatic hydrolysis of Atlantic mackerel (*Scomber scombrus*) side streams performed with the use of endopeptidase.

The novelty of this research lies in its innovative approach to improving physicochemical and functional properties of FPH recovered from Atlantic mackerel through the combined use of US and enzymatic treatment with aminopeptidase AAP. AAP is a bridged bimetallic enzyme that removes the N-terminal amino acid from a peptide chain. It has been shown to be an unusually stable enzyme, and that is why AAP was chosen in this study. AAP can maintain its activity at 70°C for several hours and is only partially inactivated in 8 M urea (26). Moreover, this enzyme has a hydrophobic active site that can better interact with hydrophobic residues such as leucine and phenylalanine (27). The present study explores the effects of both single and combined treatments of US and AAP on mackerel FPH, which is relatively novel. This dual approach aims to leverage the benefits of both methods to enhance the quality of FPH through process optimization.

## 2 Materials and methods

### 2.1 Enzymatic hydrolysis

Side streams of Atlantic mackerel (*Scomber scombrus*) were obtained from a local fish processing factory in Fosnavåg (Norway), in October 2023, and enzymatic hydrolysis was performed as described in Cropotova et al. (12). The fish side streams were minced fresh with a Hobart A 200 mincer on the day of arrival to NTNU (Ålesund, Norway), divided into 1-kilogram (kg) batches, and stored in a freezer at −30 ± 2°C for 3 weeks until hydrolysis could be performed. Prior to enzymatic hydrolysis, the fish mince (1 kg) was defrosted at 4 ± 1°C overnight and mixed with 1 kg of distilled water. It underwent enzymatic hydrolysis for 1 h at 50 ± 2°C with the endopeptidase Alcalase^®^ (Sigma-Aldrich, Germany) added in the amount of 0.1% (w/w) of the raw material weight into bioreactor. After the hydrolysis, the bones were removed from the mixture by filtering the hydrolysate through a sieve, followed by inactivation of enzyme in a microwave oven at 90°C for 10 min. After that, the mixture was cooled down up to 30°C before being transferred to 1-liter centrifugation bottles and then centrifuged at 4100 g at 4°C for 30 min resulting in three fractions: lipids (oil), fish protein hydrolysate (FPH) consisting of water-soluble peptides, and sludge (insoluble fraction). The hydrolysate fraction was separated from the rest of the fractions, placed into a − 80°C freezer for 24 h, and freeze-dried in a Labconco Freezone Console 12 L Freeze Dry System (−80°C). This mackerel hydrolysate was further used both as a control sample and for further US and enzymatic treatments with an aminopeptidase.

### 2.2 Ultrasound and enzyme treatment

Experimental FPH samples were subjected to ultrasound treatment at 300 W and 500 W with a 20 kHz probe (Sonics & Materials Inc., Danbury, CT., United States, model: VCX 1500) with and without addition of aminopeptidase from *Aeromonas proteolytica* (AAP) (EC 3.4.11.10). The ultrasonic power of 300 W and 500 W was selected based on our previous study (12), which gave promising results regarding quality improvement of mackerel FPH after US treatment. In the present study, the main goal was to study how the quality and functional properties of mackerel FPH will be affected after the same US treatment combined with AAP. In total, six samples of mackerel hydrolysates were used in the study. FPH samples were dissolved in distilled water in a ratio of 20 g FPH per 400 ML, and 1 mL of AAP solution dissolved in 10 mL distilled water was added. The 1.2-cm vibrating titanium tip of the US probe was immersed in the FPH solution followed by its irradiation with an ultrasonic wave directly from the horn tip. FPH samples were treated for 5 min with the intervals of 5 s passive (rest) and active (treatment) phase each. The temperature of FPH solutions after US treatment was 40 ± 2°C. Control and experimental FPH samples not subjected to US treatment were placed in a water bath at 40 ± 2°C for 10 min to match the temperature recorded in US-treated FPH samples. Then, all experimental FPH solutions with AAP were subjected to microwave treatment for 2 min 30 s at 800 W to reach 90°C and then kept in the microwave oven for 5 min more to inactivate the enzyme. After that, all FPH solutions were collected frozen at −80°C for 24 h prior to freeze drying (Labconco Freezone Console 12 L Freeze Dry System) for 1 week before further analyses.

### 2.3 Proximate composition analysis

The content of nitrogen (N) in the obtained FPH was determined using a Vario-El-Cube CHNS Elemental Analyzer (Elementar, GmbH, Germany). Approximately 4 mg of a dried sample was weighed out in tin capsules and oxidized at 1150°C. The amount of protein in the samples was estimated using a nitrogen-to-protein conversion factor determined for fish raw material of 6.25 (28). Water content was determined gravimetrically after drying at 105°C for 24 h. Ash content was determined by incineration to constant weight at 550°C (29). Lipid content in FPH was calculated mathematically due to a very low amount of fat in the samples through the deduction of total protein, ash, and water content from 100.

### 2.4 Soluble proteins in mackerel FPH

To determine soluble proteins in FPH samples, protein extracts were prepared by dissolving 0.1 g of each FPH sample in 10 mL of distilled water. The solutions were homogenized and centrifuged. Water-soluble proteins were determined in triplicates by using the Lowry method (30). Bovine serum albumin (BSA) was used to prepare a standard curve. The absorbance of the incubated standards and samples was determined using a SpectraMax ix3 microplate reader (Molecular Devices, United States) at a wavelength of 750 nm (30). The analyses were run in triplicate, and the mean value ±SD was calculated.

### 2.5 Thiol groups

Total thiol groups were determined according to Ellman (31) and Kvangarsnes et al. (32). To 0.1 mL of the water-soluble extract or distilled water (blank), 0.8 mL of 8 M urea and 0.1 mL of DTNB were added. Samples were mixed, incubated at room temperature for 30 min, and centrifuged for 3 min at 11000 g at room temperature. The absorbance was measured spectrophotometrically with Shimadzu UV-1800 UV/visible scanning spectrophotometer (Shimadzu Europa GmbH, Germany) at 412 nm with the blank as reference. The thiol content was calculated using a molar extinction coefficient of 14,290 M^−1^ cm^−1^. The results are expressed as nmol/mg protein.

### 2.6 Molecular weight distribution of FPH

Molecular weight distribution analysis of mackerel FPH was performed according to the method described in Cropotova et al. (12). Freeze-dried FPH was diluted with Milli-Q (MQ) water to a concentration of 10 mg/mL. Then, 100 μL of the diluted FPH solution was further diluted with 900 μL of 10% acetonitrile in MQ water in an HPLC vial. Analysis was performed on an AQUITY UPLC H-Class PLUS System (Waters Corporation, Milford, MA, United States) with an AQUITY BEH125 SEC 1.7u 4.6 mm × 150 mm column (Waters) and an AQUITY UPLC PDA Detector (Waters Corporation, Milford, MA, United States) set to 220 nm. Runs were isocratic, and a 100 mM phosphate buffer (pH 6.8) was used as the mobile phase with 0.5 mL/min of flow rate, an injection volume of 2 μL, and a total run time of 15 min. The column temperature was set to 30°C for analysis. Bovine serum albumin (66,000 Da), cytochrome C (12,327 Da), aprotinin (6,512 Da), insulin A (2,531 Da), Leu-enkephalin (555.6 Da), Met-enkephalin (573.7 Da) Val-Tyr-Val (379.5 Da), and Gly-Tyr (238.2 Da) were used as standards. All were purchased from Merck. Chromatograms were manually integrated and separated into intervals of <0.2, 0.2–0.5, 0.5–1, 1–2, 2–5, and >5 kDa, expressed as percentages of the total area. All samples were analyzed in triplicate.

### 2.7 Degree of hydrolysis

The degree of hydrolysis (DH) was analyzed by formol titration as the proportion (%) of free amino groups with regard to the total nitrogen in the sample previously determined by the CHNS method (28). A FPH sample of 1.5 g was weighed into a beaker and filled up to 50 g with distilled water. The pH was adjusted to 7.0 using 0.1 M NaOH, and then, 10 mL of 9% w/w formaldehyde with a pH of 8.5 was added. The beaker was covered with aluminium foil and stirred for 5 min. For the titration, a TITROLINE 7000 automatic titrator (SI Analytics, Xylem Analytics Germany Sales GmbH & Co. KG, Germany) was used. The titrator was rinsed three times before starting the titration. Furthermore, the titration was set to pH 8.5 with stopping automatically when reaching a pH of 8.5. The samples were titrated with 0.1 M NaOH, and the used amount of NaOH was recorded. Degree of hydrolysis was further determined as described by Cropotova et al. (12).

### 2.8 Amino acid profile

Amino acid composition analysis of mackerel FPH was performed according to the method previously described in Cropotova et al. (12). Approximately 50 mg of freeze-dried FPH was weighed into glass tubes, and 1 mL 6 M HCl was added. The glass tubes were placed into a heating cupboard for 24 h, at 105°C. Samples were diluted 50 times using distilled water before filtering through 0.22 μm.

For the derivatization, 200 μL of the sample were transferred to a glass tube, containing 600 μL 0.4 M borate buffer (pH 9). Then, 400 μL FMOC (9-fluorenylmethoxycarbonyl chloride, 15 mM in acetonitrile) was added, vortexed for 1 min, and then allowed to stand at room temperature for 4 min. After amino acid derivatization with FMOC, 400 μL ADAM (60 mM in acetonitrile:water 2:1) was added.

Amino acids were determined using a Shimadzu Nexera XR HPLC system, equipped with a PDA detector (Shimadzu, United States). Separation of amino acids was carried out on a Restec ARC-18 column (10 mm x 2.1 mm) at 30°C. The mobile phase was 0.1% formic acid with 20 mM ammonium formate and 0.1% formic acid with 10 mM ammonium formate in 90:10 acetonitrile water in gradient mode, with a flowrate of 0.8 mL/min.

### 2.9 Free amino acid profile

Water extracts were made by adding 0.1 g of FPH in 10 mL of distilled water. One mL of water extract was mixed with 0.25 mL of sulfosalicylic acid (10%) and incubated at 4°C for 30 min. The samples were centrifuged at 10000 g for 10 min. For the derivatization, 300 μL of the sample were transferred to a glass tube, containing 400 μL 0.4 M borate buffer (pH 9). Then, 100 μL FMOC (9-fluorenylmethoxycarbonyl chloride, 15 mM in acetonitrile) was added, mixed on a vortex for 1 min, and then allowed to stand at room temperature for 4 min. After amino acid derivatization with FMOC, 300 μL ADAM (60 mM in acetonitrile:water 2:1) was added. Free amino acids were determined using a Shimadzu Nexera XR HPLC system, as described in chapter 2.8.

### 2.10 Colour measurements

Colour parameters of the FPH obtained from mackerel side streams were determined using a Minolta Chromometer Model CR 400 (Konica Minolta, Japan) calibrated on a white reference plate before use. L* (lightness), a* (redness), and b* (yellowness) were measured on the protein hydrolysates in triplicate at a room temperature. The L*, a*, and b* parameters of the CIELAB scale were measured according to the lab scale established by Commission Internationale de l’Éclairage (33), and the average with standard deviation was calculated.

### 2.11 Statistical analysis

All the results were expressed as the mean ± standard deviation (s.d.), where *p*-values of < 0.05 were considered to be significant. For each analysis, three replicates were run. Statistical analyses were performed by one-way and two-way analysis of variance (ANOVA) followed by Tukey’s post-test (Statgraphics, United States) as described more in detail in Cropotova et al. (12).

## 3 Results and discussion

### 3.1 Proximate composition

The proximate composition of FPH obtained from mackerel side streams is displayed in Table 1.

**Table 1:** Proximate composition of FPH obtained from Atlantic mackerel side streams.

FPH sample	Water, %	Ash, %	Total protein content, %	Lipid content, %
Control	9.5 ± 1.3^a^	10.9 ± 0.1^a^	78.2 ± 0.2^a^	<2
Control + AAP	9.5 ± 1.3^a^	10.9 ± 0.1^a^	78.2 ± 0.2^a^	<2
300 W	9.2 ± 1.1^a^	11.7 ± 0.1^b^	78.2 ± 0.2^a^	<1
300 W + AAP	9.2 ± 1.1^a^	11.7 ± 0.0^b^	78.2 ± 0.2^a^	<1
500 W	9.1 ± 1.2^a^	11.8 ± 0.2^b^	78.2 ± 0.2^a^	<1
500 W + AAP	9.1 ± 1.2^a^	11.8 ± 0.1^b^	78.2 ± 0.2^a^	<1

Mean values ± standard deviation are shown. Different letters within the same column indicate significant differences, *p* < 0.05.

No significant differences (*p* < 0.05) in water, ash, and total protein content were found between the samples (Table 1). This can be because all experimental FPH samples were obtained from the control sample of mackerel hydrolysate by either enzyme modification reaction or both enzyme and US treatment and were all subjected to freeze drying at the same conditions. When compared to our previous research on US treatment of FPH obtained from Atlantic mackerel by enzymatic hydrolysis in 2022 (12), it can be observed that FPH described in the current study is characterized by significantly higher water and ash content together with lower fat and protein content. This tendency can be explained by a variation of protein, water, and fat content in the fish muscle during the season and in different years, which affects the proximate composition of the obtained FPH.

### 3.2 Soluble proteins in mackerel FPH

According to the results displayed in Table 2, the amount of soluble proteins increased significantly (*p* < 0.05) in FPH sample treated with AAP compared to control. This tendency can be because AAP breaks down bigger peptides of FPH into smaller peptide units, exposing the protein’s hydrophilic site. The AAP-treated FPH with more exposed hydrophilic sites possess higher solubility since they are able to form hydrogen bonds with water (34). However, protein solubility decreased significantly (*p* < 0.05) after application of US treatment of FPH both with and without use of AAP compared to control sample. This can be explained by the cavitation effect of US treatment generated by high local pressure and temperature of ultrasonic waves, resulting in aggregation and denaturation of peptides and decreasing their solubility (12). The detrimental effect of US treatment at 300 W resulted also in significant (*p* < 0.05) drop in soluble protein in FPH after use of AAP, while at 500 W the changes are not significant. This can be explained by cavitation and mechanical oscillation effect of ultrasound altering the enzyme and the substrate characteristics (35). Thus, US treatment could modify the conformation of AAP and characteristics of the substrate, affecting the reaction between the enzyme and the FPH substrates. However, in our previous research on US treatment of mackerel hydrolysates (12), a significant increase in protein solubility was observed along with an increase in power of ultrasonication for all FPH samples. This tendency can be explained by the fact that in the present study, we used higher concentration of mackerel hydrolysate for US treatment (20 g/400 mL) compared to the previous study (10 g/400 mL) (12), which could result in increased frequency of molecular collisions during ultrasonication, thereby promoting aggregation or denaturation of peptides (35).

**Table 2:** Soluble proteins in mackerel FPH samples.

FPH samples	Soluble protein, %	Thiol groups, nmol/mg
Control	84.4 ± 0.81^a^	12.86 ± 0.77^a^
Control+AAP	91.13 ± 1.32^b^	10.33 ± 1.34^b^
300 W	79.1 ± 1.28^c^	9.34 ± 0.65^b^
300 W + AAP	50.27 ± 0.7^d^	5.85 ± 0.85^c^
500 W	70.33 ± 1.85^e^	9.54 ± 0.36^b^
500 W + AAP	72.47 ± 0.58^e^	6.77 ± 0.57^c^

Mean values ± standard deviation are shown. Statistical analysis was performed by two-way ANOVA. Different letters within the same column indicate significant differences, *p* < 0.05.

### 3.3 Thiol groups

Protein oxidation of mackerel FPH with and without US treatment and use of AAP were assessed in terms of thiol groups. According to the results displayed in Table 2, there was a significant decrease (*p* < 0.05) in thiol groups in all experimental FPH samples compared to control. This phenomenon can be explained by the ultrasound-induced cavitation and mechanical oscillation effect unfolding secondary and tertiary structures of peptides in FPH samples. This further results in the reduction of total thiol (-SH) groups due the exposure of amino acid residues prone to oxidation to the surrounding environment (36). Similar findings were observed in our previous study on US treatment of mackerel hydrolysates (12) when ultrasonication of FPH resulted in significant decrease in thiol groups for US-treated samples at 300 W and 450 W. However, in the present study, there was no significant difference in thiol groups between Control+AAP and US-treated FPH samples at 300 W and 500 W without the use of AAP, while it was a big and significant drop in total thiols between 300 W and 300 W + AAP, as well as 500 W and 500 W + AAP. We hypothesize that AAP acting on US-induced unfolded secondary and tertiary structures of peptides more actively oxidizes the available thiol groups that were previously located inside the folded peptides and are now exposed to the surface to disulfide bonds (37).

### 3.4 Molecular weight distribution of FPH

**Figure 1:**
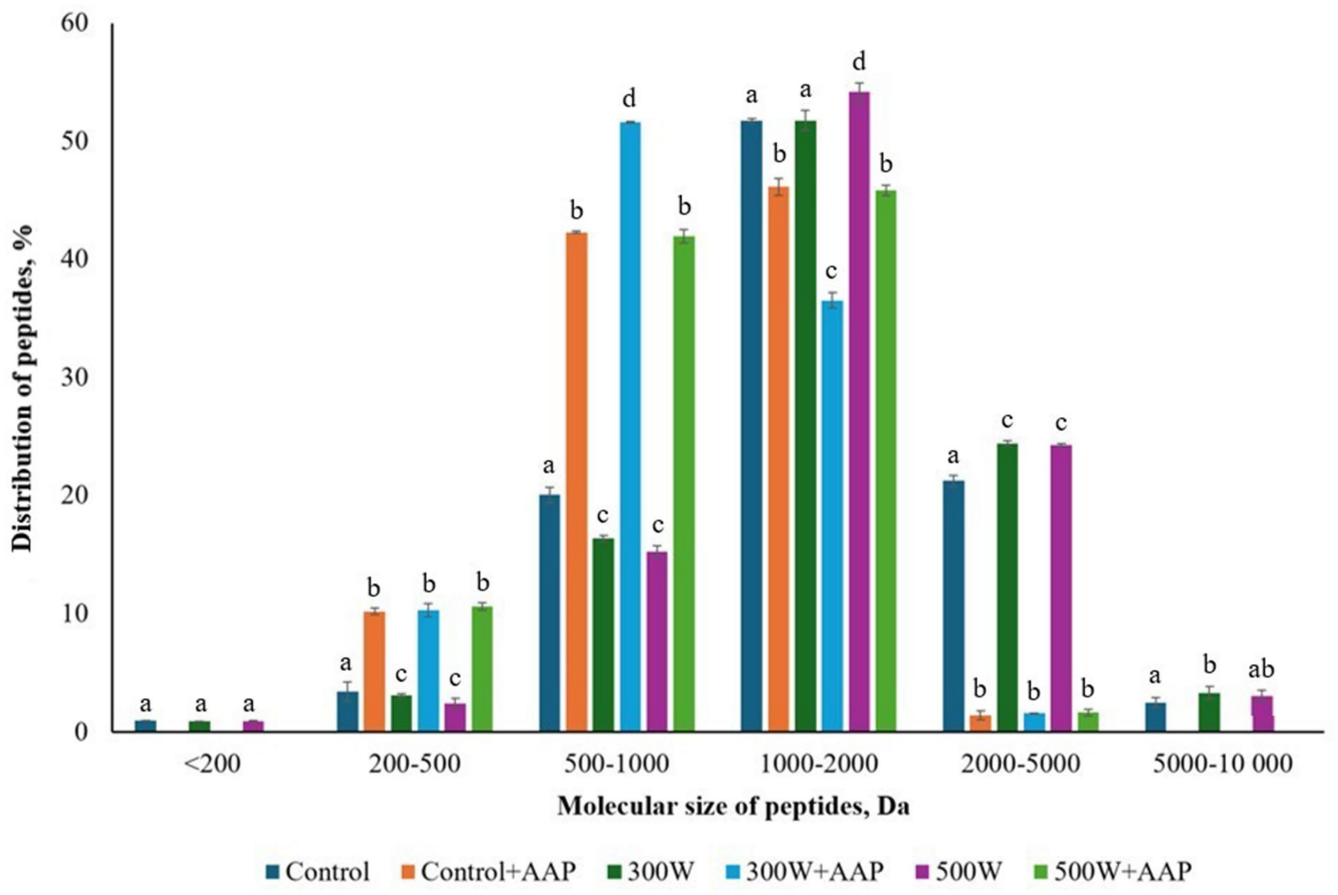
Molecular weight distribution in mackerel FPH. All the datasets have been analyzed by one-way ANOVA. (*) *p* < 0.05.

The MWD of mackerel FPH (Figure 1) revealed that the use of AAP caused a significant (*p* < 0.05) increase in the distribution of small peptides with MW between 200 and 1,000 Da in all FPH samples compared to FPH without AAP. At the same time, there was a significant drop in the proportion of medium-size peptides with MW of 1,000–5,000 Da in all FPH with AAP compared to FPH without AAP. Moreover, the use of AAP reduced the proportion of medium-size peptides with MW of 2000–5,000 Da by approximately 94% in all FPH compared to FPH without AAP. In addition, no medium-size peptides with MW of 5,000–10,000 Da were found in FPH treated with AAP, suggesting that the use of this enzyme resulted in smaller peptide molecules by hydrolyzing N-terminal amino acid residues from polypeptides (38). It is worth mentioning that the molecular weight of peptides is associated with bitter taste of FPH (39). Thus, bitterness of FPH is mainly ascribed to small peptides of less than 1,000 Da (40). In the present study, US treatment of FPH at 300 W and 500 W significantly reduced the distribution of small peptides in the MW range of 200–1,000 Da compared to control. However, as mentioned above, the use of AAP alone and in combination with ultrasonication significantly increased the amount of small peptides in the MW range of 200–1,000 Da compared both to control and to US-treated FPH samples. This suggests that ultrasonication of FPH applied without use of AAP may lead to a significant reduction of bitterness due to a significant reduction of the proportion of small peptides, while the use of AAP gives the opposite results. According to Figure 1, US-treated FPH at 300 W and 500 W had significantly higher proportion of medium-size peptides with MW 2000–5,000 Da compared to control. This phenomenon can be explained by the fact that US treatment could result in aggregation of the previously US-cleaved small peptides colliding with each other and forming macromolecular aggregate fragments through intermolecular interactions (41).

### 3.5 Degree of hydrolysis

**Figure 2:**
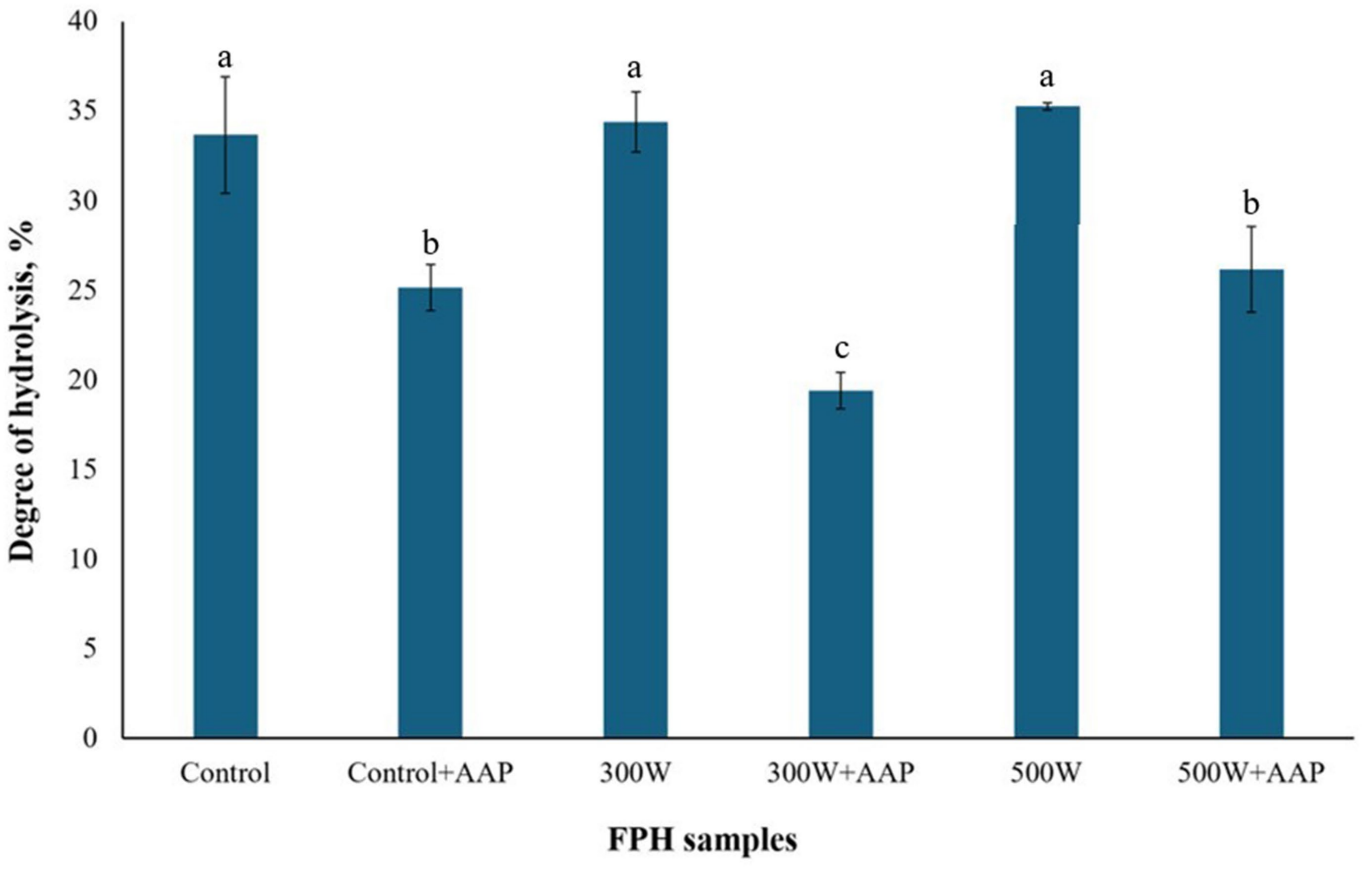
Degree of hydrolysis of mackerel PFH. All the datasets have been analyzed by one-way ANOVA. (*) *p* < 0.05.

According to Figure 2 showing the results of degree of hydrolysis (DH), there was a small but insignificant increase in DH in US-treated FPH at 300 W and 500 W compared to control. This phenomenon can be explained by a small cavitation effect causing fragmentation and denaturation of peptides, including also breaking down the polypeptide molecules into smaller pieces (42), which increases the amount of free amino acid groups and affects the degree of hydrolysis (12). In our previous study on US treatment of FPH from mackerel, the increase in DH of FPH after ultrasonication at 300 W and 450 W compared to control was also small but significant (12). We suggest that this difference in DH of mackerel hydrolysates between the two US treatment experiments is related to the cavitation effect of turbulent forces, when micro-streaming of more concentrated solution of protein hydrolysates results in higher speed of collision and aggregation of peptides (35). This also supports our previous suggestion about cavitation effect resulting in denaturation of peptides and their further fragmentation into smaller molecules, which is in agreement with MWD data displayed in Figure 1. However, the use of AAP significantly reduced DH in all AAP-treated FPH compared to control and AAP-untreated FPH. The largest drop in DH was observed for the sample 300 W + AAP, while there was no significant difference in DH between Control+AAP and 500 W + AAP. This suggests that the use of AAP resulted in a reduction of DH in FPH samples. We hypothesize that AAP hydrolyzed N-terminal amino acid residues from medium-size peptides (38), resulting in smaller peptide fragments which further clustered together and aggregated under thermal (Control + AAP) and US treatment (300 W + AAP and 500 W + AAP) (41), reducing DH of FPH.

### 3.6 Amino acid profile

According to Table 3 showing the results of the total amino acid profile of mackerel FPH, the most abundant amino acids in FPH samples were glycine, glutamic acid, and proline. The predominance of these amino acids in the amino acid composition of FPH can be explained by the high content of fish skin in the raw material (mackerel side streams) used to produce the FPH. This is in accordance with the results from our previous research on US treatment of mackerel FPH (12), where raw material contained a high content of fish skin, and the most abundant amino acid was also glycine. The treatment of FPH with AAP increased the proportion of aspartic and glutamic acids, glycine, proline, phenylalanine, and isoleucine compared to AAP-untreated FPH. At the same time, the use of this enzyme resulted in a significant (*p* < 0.05) decrease in the proportion of alanine, valine, and tyrosine. A decrease in lysine was also observed when using AAP in combination with US treatment. The proportion of the latter dropped drastically up to 70–81% after the treatment of FPH with AAP (with and without use of ultrasound). Normally, tyrosine residues are partially or fully buried in the protein structure and are not present on the surface of protein molecules for enzymatic reaction by AAP (43). However, in the present study, we hypothesize that enzymatic hydrolysis previously performed by alcalase cleaved protein molecules in a way that further cleave N-terminal region of tyrosine, as well as alanine, valine, and lysine of the region-selective peptides of FPH samples by AAP became achievable. The proportion of essential amino acids increased in all experimental FPH samples except 500 W + AAP compared to control, but the increase was not significant. However, the increase suggests a positive influence of both ultrasound and AAP treatment on nutritional profile of mackerel hydrolysates.

**Table 3:** Amino acid composition in mackerel FPH, % of total amino acids.

Amino acids	Control	Control + AAP	300 W	300 W + AAP	500 W	500 W + AAP
Arginine	6.29 ± 0.22^a^	8.05 ± 0.14^b^	6.12 ± 0.22^a^	6.84 ± 0.05^c^	6.33 ± 0.21^a^	5.98 ± 0.08^a^
Serine	4.41 ± 0.15^a^	4.28 ± 0.06^a^	6.1 ± 0.35^b^	4.97 ± 0.06^c^	6.41 ± 0.21^b^	4.25 ± 0.02^a^
Aspartic acid	6.88 ± 0.25^a^	7.38 ± 0.07^ab^	5.21 ± 0.65^c^	7.87 ± 0.33^ad^	4.88 ± 0.41^c^	8.3 ± 0.15^bd^
Glutamic acid	9.54 ± 0.29^ad^	10.82 ± 0.12^ac^	9.16 ± 1.08^ad^	12.96 ± 0.73^b^	8.87 ± 0.8^d^	11.87 ± 0.17^bc^
L-threonine	3.84 ± 0.1^a^	3.42 ± 0.25^a^	5.51 ± 0.33^b^	4.15 ± 0.57^a^	5.48 ± 0.04^b^	3.5 ± 0.25^a^
Glycine	23.69 ± 0.73^a^	24.14 ± 0.14^a^	22.05 ± 0.66^b^	26.58 ± 0.6^c^	22.07 ± 0.41^b^	26.81 ± 0.31^c^
Alanine	6.43 ± 0.24^a^	3.41 ± 0.04^bc^	6.07 ± 0.48^a^	3.03 ± 0.14^b^	6.02 ± 0.63^a^	4.14 ± 0.08^c^
Proline	9.65 ± 0.3^a^	11.2 ± 0.16^b^	5.86 ± 0.14^ce^	6.56 ± 0.1^d^	6.32 ± 0.24^de^	12.33 ± 0.18^f^
Methionine	1.6 ± 0.03^a^	1.04 ± 0.1^bd^	0.54 ± 0.31^c^	1.27 ± 0.04^ad^	0.55 ± 0.14^c^	1.25 ± 0.07^ad^
Valine	4.86 ± 0.24^a^	3.5 ± 0.66^bce^	3.94 ± 0.72^acd^	2.49 ± 0.04^b^	5.17 ± 0.88^ae^	2.35 ± 0.2^bd^
Phenylalanine	0.97 ± 0.26^a^	4.04 ± 0.18^b^	1.23 ± 0.36^a^	4.84 ± 0.28^c^	1.01 ± 0.17^a^	3.33 ± 0.11^d^
Isoleucine	2.81 ± 0.16^a^	5.29 ± 0.28^b^	2.44 ± 0.25^a^	6.07 ± 0.34^c^	2.58 ± 0.06^a^	4.74 ± 0.16^b^
Leucine	6.09 ± 0.79^a^	6.14 ± 0.18^a^	5.36 ± 2.68^a^	5.16 ± 0.08^a^	6.47 ± 0.32^a^	5.54 ± 0.82^a^
Cystine	0.17 ± 0.01^a^	0.41 ± 0.18^b^	0.47 ± 0.09^b^	0.36 ± 0.02^ab^	0.25 ± 0.04^ab^	0.28 ± 0.01^ab^
Histidine	2.23 ± 0.46^ab^	1.89 ± 0.59^a^	3.15 ± 0.42^b^	2.59 ± 0.7^ab^	3.61 ± 0.33^b^	2.14 ± 0.13^ab^
Lysine	3.3 ± 0.24^a^	2.84 ± 0.21^a^	6.91 ± 0.7^b^	1.74 ± 0.44^c^	3.74 ± 0.39^a^	1.36 ± 0.14^c^
Tyrosine	7.23 ± 0.29^a^	2.12 ± 0.07^bf^	9.87 ± 0.76^c^	2.53 ± 0.38^bdf^	10.24 ± 1.14^ce^	1.86 ± 0.2^f^
Essential	25.71 ± 2.0^ab^	28.17 ± 0.22^a^	29.09 ± 2.7^a^	28.3 ± 0.37^a^	28.61 ± 0.68^a^	24.2 ± 0.45^b^

Mean values ± standard deviation are shown. Different letters indicate significant differences (*p* < 0.05).

### 3.7 Free amino acid profile

The FAA profile of mackerel FPH is shown in Table 4.

**Table 4:** Free amino acid composition in mackerel FPH, % of total free amino acids.

Amino acids	Control	Control + AAP	300 W	300 W + AAP	500 W	500 W + AAP
Arginine	2.94 ± 0.59^a^	9.46 ± 0.35^bc^	10.3 ± 0.47^b^	9.76 ± 0.61^bc^	10.44 ± 0.92^b^	8.66 ± 0.45^c^
Asparagine	3.23 ± 0.19^a^	16.67 ± 0.32^b^	6.25 ± 1.5^c^	13.69 ± 0.78^d^	7.82 ± 1.63^c^	11.76 ± 0.51^d^
Glutamine	3.15 ± 0.16^a^	1.18 ± 0.05^b^	2.75 ± 1.06^a^	0.8 ± 0.04^b^	2.88 ± 0.57^a^	0.83 ± 0.16^b^
Serine	9.40 ± 1.29^a^	1.66 ± 0.1^b^	2.65 ± 0.42^b^	2.66 ± 0.21^b^	2.61 ± 0.76^b^	2.12 ± 0.06^b^
Aspartic acid	2.16 ± 0.29^ad^	1.48 ± 0.11^be^	2.45 ± 0.24^c^	1.95 ± 0.12^a^	2.35 ± 0.16^cd^	1.75 ± 0.05^ae^
Glutamic acid	3.06 ± 0.24^a^	3.45 ± 0.17^a^	4.38 ± 0.24^b^	4.31 ± 0.27^b^	4.04 ± 0.13^b^	3.87 ± 0.08^b^
Threonine	1.67 ± 0.22^a^	1.43 ± 0.07^ace^	1.25 ± 0.05^bc^	2.05 ± 0.15^d^	1.25 ± 0.09^be^	1.68 ± 0.04^a^
Glycine	5.43 ± 0.45^a^	8.38 ± 0.44^bc^	9.62 ± 0.27^bcd^	10.15 ± 1.03^de^	9.77 ± 0.37^ce^	8.68 ± 0.19^bde^
Alanine	4.72 ± 0.07^a^	4.62 ± 0.07^a^	3.32 ± 0.16^b^	5.27 ± 0.43^ac^	3.68 ± 0.5^b^	5.69 ± 0.15^c^
Proline	7.45 ± 0.97^a^	9.12 ± 0.39^bd^	11.55 ± 0.67^c^	5.7 ± 3.42^ade^	11.1 ± 0.46^c^	8.75 ± 0.22^abe^
Methionine	3.59 ± 0.38^a^	2.11 ± 0.04^b^	2.73 ± 0.06^bcde^	2.97 ± 0.2^ad^	3.01 ± 0.09^ac^	3.2 ± 0.42^ae^
Valine	4.56 ± 0.58^a^	2.6 ± 0.12^b^	3.57 ± 0.22^ab^	2.91 ± 0.32^b^	3.99 ± 0.07^ab^	4.32 ± 1.04^a^
Phenylalanine	3.44 ± 0.25^ab^	4.69 ± 1.94^a^	2.03 ± 0.1^ab^	4.88 ± 0.68^ab^	2.45 ± 0.18^b^	2.86 ± 0.38^a^
Isoleucine	2.74 ± 0.22^acd^	4.85 ± 2.12^be^	2.82 ± 0.13^abd^	6 ± 1.83^ce^	2.97 ± 0.19^ab^	3.52 ± 0.09^de^
Leucine	11.92 ± 0.22^a^	9.06 ± 1.09^b^	11.05 ± 0.44^ac^	9.19 ± 0.61^b^	12.12 ± 0.17^a^	9.96 ± 0.16^bc^
Cystine	1.05 ± 0.23	nd	nd	nd	nd	nd
Histidine	11.28 ± 0.76^ac^	16.61 ± 2.32^b^	7.17 ± 1.86^ad^	15.19 ± 1.61^bc^	5.55 ± 2.07^d^	18.17 ± 1.01^b^
Lysine	11.22 ± 0.98^a^	1.33 ± 0.5^b^	9.91 ± 1.56^a^	1.43 ± 0.49^b^	9.74 ± 1.46^a^	2.87 ± 0.49^b^
Tyrosine	7 ± 0.26^a^	1.28 ± 0.11^b^	6.2 ± 1.99^a^	1.12 ± 0.13^b^	4.23 ± 0.37^c^	1.3 ± 0.32^b^
Bitter FAA*	38.42 ± 2.51^a^	37.05 ± 3.32^a^	37.08 ± 1.19^a^	36.9 ± 4.37^a^	39.32 ± 0.61^a^	38.31 ± 1.52^a^

* Free amino acids giving bitterness: methionine, phenylalanine, valine, isoleucine, leucine, and proline (15). Mean values ± standard deviation are shown. Different letters indicate significant differences (*p* < 0.05).

There was a significant increase in arginine, asparagine, and glycine in all experimental FPH samples compared to control. Arginine and glycine are considered conditionally essential amino acids which are normally not essential, except in times of illness and stress (44). However, their adequate amounts in the diet are very important under stress or body weakness conditions. Arginine, as a key substrate for nitric oxide synthase, is responsible for the production of nitric oxide, which is a very important compound for blood pressure regulation and cardiovascular health. In addition, arginine enhances the immune function of T cells and supports wound healing by promoting collagen synthesis and cell proliferation (44). Glycine, being a major component of collagen, also takes part in its synthesis, supporting skin, joint, and connective tissue health. Moreover, glycine contributes to neurological balance and sleep regulation by acting as an inhibitory neurotransmitter in the central nervous system (44). Therefore, the significant increase in arginine (66–72%), asparagine (48–81%), and glycine (35–47%) in all FPH samples compared to control after AAP and US treatment represents a positive factor. However, at the same time, both the use of AAP and US treatment led to a significant decrease in serine and cystine. Proportion of lysine and tyrosine has also dropped after AAP and US treatment but insignificantly. Serine and lysine have shown antioxidative effects (45), and in our study, the drop in the free amino content of serine in all experimental FPH was the highest (72–83%) compared to control. The reduction in free lysine content was also the biggest for FPH samples (300 W + AAP (88%), 500 W (87%), and 500 W + AAP (74%)). The proportion of tyrosine also decreased drastically in these FPH samples, as follows: 300 W + AAP (82%), 500 W (84%), and 500 W + AAP (81%). However, there was no significant difference in the proportion of hydrophobic free amino acids responsible for bitter taste (15) between control and experimental FPH samples in our study. There are many factors influencing this variation in FAA. The FAA profile is known to differ due to enzyme used, the time, and the degree of hydrolysis (46). In addition, the US treatment generates heat and cavitation effect, affecting the conformation and structure of the peptides. This explains why the liberation of free amino acids was affected in different ways in experimental FPH samples, thereby affecting the differences in free amino acid distribution. However, more research is needed to understand why the proportion of the specific FAA changed after both AAP and US treatment.

### 3.8 Color parameters of mackerel FPH

Color parameters represent one of the key sensory attributes in consumer acceptability and successful commercialization of protein hydrolysates. Previous studies have shown that lighter-colored FPH are better perceived by consumers because they are commonly associated with freshness compared to darker FPH (47).

According to the results of color parameters of mackerel FPH displayed in Table 5, there was a significant increase in lightness (L*-values) for FPH samples treated at 300 W, 300 W + AAP, and 500 W compared to control. At the same time, it was a significant drop in redness (a*-values) in FPH treated at 300 W and 500 W compared to control. The same effect of US treatment on lightness and redness of FPH extracted from Atlantic mackerel side streams was observed in our previous study (12). We hypothesize that this effect is related to the changes in secondary structure of peptide molecules due to the cavitation effect of US treatment resulting in a shift of the absorption peaks of light toward higher wavelengths increasing lightness of FPH (48). However, at the same time, the use of AAP led to a significant (*p* < 0.05) drop in lightness and increase in redness in all experimental FPH compared to AAP-untreated samples. Taking into account the severe drop in free tyrosine content in all AAP-treated FPH samples (Table 4), we hypothesize that the darker color of these hydrolysates is associated with oxidation of tyrosine (49). Regarding yellowness of FPH, a significant (*p* < 0.05) increase in b*-values was observed only for FPH samples after US treatment at 300 W and 500 W compared to control. This phenomenon is probably related to the US-induced oxidation of lipids rich in polyunsaturated fatty acids which are present in FPH samples (Table 1) due to high temperatures and micro-streaming/turbulence eddies formed during cavitation (50), as well as free radicals generated by sonication in the aqueous phase during cavity collapses (51).

**Table 5:** Color parameters of mackerel FPH.

Color parameters	Control	Control + AAP	300 W	300 W + AAP	500 W	500 W + AAP
L*-value	52.93 ± 0.98^a^	50.75 ± 0.18^b^	61.05 ± 0.35^d^	54.49 ± 0.30^c^	60.30 ± 0.66^d^	52.85 ± 1.07^a^
a*-value	0.78 ± 0.40^a^	1.24 ± 0.36^b^	−2.06 ± 0.03^c^	0.15 ± 0.09^d^	−0.78 ± 0.19^e^	1.28 ± 0.38^b^
b*-value	17.07 ± 1.31^a^	18.68 ± 1.31^a^	20.26 ± 0.22^b^	19.25 ± 0.73^ab^	19.76 ± 0.18^ab^	20.72 ± 0.63^b^

Mean values ± standard deviation are shown. Statistical analysis was performed by two-way ANOVA. Different letters indicate significant differences.

## 4 Conclusion

The study has shown that physicochemical characteristics of FPH obtained from Atlantic mackerel side streams can be effectively improved through both single and combined post-treatments involving ultrasonication at 300 W and 500 W and enzymatic treatment with aminopeptidase AAP. However, the application of US treatment, both alone and in combination with AAP, resulted in a significant decrease in functionality of FPH expressed through amount of soluble proteins in all experimental FPH samples compared to control due to peptide aggregation caused by cavitation effect and turbulence eddies. In addition, the use of AAP resulted in increased distribution of small peptides and decreased proportion of medium-sized peptides in all AAP-treated FPH samples compared to control. Moreover, the nutritional profile of FPH was significantly enhanced by increasing the proportion of essential amino acids in all but 500 W + AAP samples compared to control. Despite these improvements, there was no significant difference in the proportion of hydrophobic free amino acids responsible for bitterness. However, ultrasonication significantly enhanced color characteristics of FPH samples US-treated at 300 W and 500 W by increasing their lightness and reducing redness. Overall, the combined use of AAP and US treatment shows promise in improving quality parameters of mackerel hydrolysates, making them more attractive for food applications. Further investigations on the use of different enzymes together with higher powers of ultrasonication as post-treatment of fish hydrolysates should be performed to improve quality parameters of FPH.

## Data Availability

The raw data supporting the conclusions of this article will be made available by the authors, without undue reservation.

## References

[1] United Nations Report. Population. Available online at: https://www.un.org/en/global-issues/population (accessed on Oct 08, 2024).

[2] Venugopal V Sasidharan A. Functional proteins through green refining of seafood side streams. Front Nutr. (2022) 9:1896. doi: 10.3389/fnut.2022.974447, PMID: 36091241 PMC9454818

[3] Salter AM Lopez-Viso C. Role of novel protein sources in sustainably meeting future global requirements. Proc Nutr Soc. (2021) 80:186–94. doi: 10.1017/S0029665121000513, PMID: 33494845

[4] Pyz-Łukasik R Chałabis-Mazurek A Gondek M. Basic and functional nutrients in the muscles of fish: a review. Int J Food Prop. (2020) 23:1941–50. doi: 10.1080/10942912.2020.1828457

[5] Skałecki P Florek M Kaliniak A. Value in use of fishes from polish aquaculture. J Anim Sci Biotechnol. (2017) 35:27–36. doi: 10.24326/jasbbx.2017.4.3

[6] Siddiqui SA Schulte H Pleissner D Schönfelder S Kvangarsnes K Dauksas E. Transformation of seafood side-streams and residuals into valuable products. Food Secur. (2023) 12:422. doi: 10.3390/foods12020422, PMID: 36673514 PMC9857928

[7] Senadheera TRL Hossain A Shahidi F. Marine bioactives and their application in the food industry: a review. Appl Sci. (2023) 13:12088. doi: 10.3390/app132112088

[8] Cruz-Casas DE Aguilar CN Ascacio-Valdés JA Rodríguez-Herrera R Chávez-González ML Flores-Gallegos AC. Enzymatic hydrolysis and microbial fermentation: the most favorable biotechnological methods for the release of bioactive peptides. Food Chem (Oxf). (2021) 3:100047. doi: 10.1016/j.fochms.2021.100047, PMID: 35415659 PMC8991988

[9] Siddik MAB Howieson J Fotedar R Partridge GJ. Enzymatic fish protein hydrolysates in finfish aquaculture: a review. Rev Aquac. (2021) 13:406–30. doi: 10.1111/raq.12481

[10] Zamora-Sillero J Ramos P Monserrat JM Prentice C. Evaluation of the antioxidant activity in vitro and in hippocampal HT-22 cells system of protein hydrolysates of common carp (*Cyprinus carpio*) by-product. J Aquat Food Prod Technol. (2017) 27:21–34. doi: 10.1080/10498850.2017.1390027

[11] Halim NRA Yusof HM Sarbon NM. Functional and bioactive properties of fish protein hydrolysates and peptides: a comprehensive review. Trends Food Sci Technol. (2016) 51:24–33. doi: 10.1016/j.tifs.2016.02.007

[12] Cropotova J Kvangarsnes K Rustad T Stangeland J Roda G Fanzaga M. Effect of ultrasound treatment on quality parameters and health promoting activity of fish protein hydrolysate (FPH) extracted from side streams of Atlantic mackerel (*Scomber scombrus*). Front Nutr. (2024) 11:1446485. doi: 10.3389/fnut.2024.1446485, PMID: 39296503 PMC11408299

[13] Kristinsson HG Rasco BA. Fish protein hydrolysates: production, biochemical, and functional properties. Crit Rev Food Sci Nutr. (2000) 40:43–81. doi: 10.1080/10408690091189266, PMID: 10674201

[14] Akimova D Kakimov A Suychinov A Urazbayev Z Zharykbasov Y Ibragimov N. Enzymatic hydrolysis in food processing: biotechnological advancements, applications, and future perspectives. Potr S J F Sci. (2024) 18:347–65. doi: 10.5219/1962

[15] Pérez-Santaescolástica C Carballo J Fulladosa E Munekata P Campagnol PB Gómez B. Influence of high-pressure processing at different temperatures on free amino acid and volatile compound profiles of dry-cured ham. Food Res Int. (2019) 116:49–56. doi: 10.1016/j.foodres.2018.12.039 30716972

[16] Nandan A Nampoothiri KM. Therapeutic and biotechnological applications of substrate specific microbial aminopeptidases. Appl Microbiol Biotechnol. (2020) 104:5243–57. doi: 10.1007/s00253-020-10641-9, PMID: 32342144 PMC7186005

[17] Idowu AT Benjakul S. Bitterness of fish protein hydrolysate and its debittering prospects. J Food Biochem. (2019) 43:e12978. doi: 10.1111/jfbc.12978, PMID: 31489658

[18] Saha BC Hayashi K. Debittering of protein hydrolyzates. Biotechnol Adv. (2001) 19:355–70. doi: 10.1016/S0734-9750(01)00070-2, PMID: 14538072

[19] Tang Y Debnath T Choi E-J Kim YW Ryu JP Jang S. Changes in the amino acid profiles and free radical scavenging activities of *Tenebrio molitor* larvae following enzymatic hydrolysis. PLoS One. (2018) 13:e0196218. doi: 10.1371/journal.pone.0196218, PMID: 29727456 PMC5935390

[20] Hunsakul K Laokuldilok T Sakdatorn V Klangpetch W Brennan CS Utama-ang N. Optimization of enzymatic hydrolysis by alcalase and flavourzyme to enhance the antioxidant properties of jasmine rice bran protein hydrolysate. Sci Rep. (2022) 12:12582. doi: 10.1038/s41598-022-16821-z, PMID: 35869265 PMC9307646

[21] Raeesi R Shabanpour B Pourashouri P. Quality evaluation of produced silage and extracted oil from rainbow trout (*Oncorhynchus mykiss*) wastes using acidic and fermentation methods. Waste Biomass Valori. (2021) 12:4931–42. doi: 10.1007/s12649-020-01331-8

[22] Meidell LS Slizyte R Mozuraityte R Carvajal AK Rustad T Falch E. Valorization of Saithe (*Pollachius virens*) residuals into protein hydrolysates — Silaging as preservation technology. Food Secur. (2024) 13:2133. doi: 10.3390/foods13132133, PMID: 38998639 PMC11241758

[23] Hassoun A Cropotova J Trollman H Jagtap S Garcia-Garcia G Parra-López C. Use of industry 4.0 technologies to reduce and valorize seafood waste and by-products: a narrative review on current knowledge. Curr Res Food Sci. (2023) 6:100505. doi: 10.1016/j.crfs.2023.100505, PMID: 37151380 PMC10160358

[24] Al Khawli F Ferrer E Berrada H Barba FJ Pateiro M Domínguez R. Innovative green Technologies of Intensification for valorization of seafood and their by-products, mar. Drugs. (2019) 17:689. doi: 10.3390/md17120689 PMC6950251 31817754

[25] Misir GB Koral S. Effects of ultrasound treatment on structural, chemical and functional properties of protein hydrolysate of rainbow trout (*Oncorhynchus mykiss*) by-products. Ital J Food Sci. (2019) 31:205–23. doi: 10.14674/IJFS-1218

[26] Chen G Edwards T D’Souza VM Holz RC. Mechanistic studies on the aminopeptidase from Aeromonas proteolytica: a two-metal ion mechanism for peptide hydrolysis. Biochemist. (1997) 36:4278–86. doi: 10.1021/bi9618676, PMID: 9100023

[27] Bienvenue DL Mathew RS Ringe D Holz RC. The aminopeptidase from *Aeromonas proteolytica* can function as an esterase. J Biol Inorg Chem. (2002) 7:129–35. doi: 10.1007/s007750100280, PMID: 11862549

[28] ISO 5983-2:2009. Determination of nitrogen content and calculation of crude protein content. Geneva, Switzerland: International Organization for Standardization (2009).

[29] AOAC. Official methods of analysis: official method for ash. In: Method no. 936.03. Washington, DC, USA: Association of Official Analytical Chemists (2000)

[30] Lowry OH Rosebrough NJ Lewis Farr A Randall RJ. Protein measurement with the Folin phenol reagent. J Biol Chem. (1951) 193:265–75. doi: 10.1016/S0021-9258(19)52451-6, PMID: 14907713

[31] Ellman GL. Tissue sulfhydryl groups. Arch Biochem Biophys. (1959) 82:70–7. doi: 10.1016/0003-9861(59)90090-6, PMID: 13650640

[32] Kvangarsnes K Dauksas E Tolstorebrov I Rustad T Bartolomei M Xu R. Physicochemical and functional properties of rainbow trout (*Oncorhynchus mykiss*) hydrolysate. Heliyon. (2023) 9:e17979. doi: 10.1016/j.heliyon.2023.e17979, PMID: 37449127 PMC10336833

[33] CIE. Commission Internationale de l’Eclairage, Improvement to Industrial Color-Difference Evaluation. Wien, Austria: Central Bureau of the CIE. (2001).

[34] Dinakarkumar Y Krishnamoorthy S Margavelu G Ramakrishnan G Chandran M. Production and characterization of fish protein hydrolysate: effective utilization of trawl by-catch. Food Chem Adv. (2022) 1:100138. doi: 10.1016/j.focha.2022.100138

[35] Huang G Chen S Dai C Sun L Sun W Tang Y. Effects of ultrasound on microbial growth and enzyme activity. Ultrason Sonochem. (2017) 37:144–9. doi: 10.1016/j.ultsonch.2016.12.018, PMID: 28427617

[36] Pathak R Bhangu SK Martin GJO Separovic F Ashokkumar M. Ultrasound-induced protein restructuring and ordered aggregation to form amyloid crystals. Eur Biophys J. (2022) 51:335–52. doi: 10.1007/s00249-022-01601-4, PMID: 35576075 PMC9233657

[37] Esteghlal S Gahruie HH Niakousari M Barba FJ Bekhit AE-D Mallikarjunan K. Bridging the knowledge gap for the impact of non-thermal processing on proteins and amino acids. Food Secur. (2019) 8:262. doi: 10.3390/foods8070262, PMID: 31319521 PMC6678513

[38] Chen SL Marino T Fang WH Russo N Himo F. Peptide hydrolysis by the binuclear zinc enzyme aminopeptidase from *Aeromonas proteolytica*: a density functional theory study. J Phys Chem B. (2008) 112:2494–500. doi: 10.1021/jp710035j, PMID: 18247603

[39] Kotsoni E Daukšas E Aas GH Rustad T Tiwari B Cropotova J. Effect of high-pressure pretreatment on enzymatic hydrolysis of a mixture of rainbow trout (*Oncorhynchus mykiss*) and Atlantic salmon (*Salmo salar*) rest raw material. Front Sustain Food Syst. (2024) 8:1313975. doi: 10.3389/fsufs.2024.1313975 PMC11205082 38921572

[40] Remme JF Korsnes S Steen S Durand R Kvangarsnes K Stangeland J. The effects of enzymes, species, and storage of raw material on physicochemical properties of protein hydrolysates from whitefish heads. Mar Drugs. (2023) 21:587. doi: 10.3390/md21110587, PMID: 37999411 PMC10671905

[41] Wang Y Li B Guo Y Liu C Liu J Tan B. Effects of ultrasound on the structural and emulsifying properties and interfacial properties of oxidized soybean protein aggregates. Ultrason Sonochem. (2022) 87:106046. doi: 10.1016/j.ultsonch.2022.106046, PMID: 35636156 PMC9149199

[42] Wang S Wang J Xue F Li C. Effects of heating or ultrasound treatment on the enzymolysis and the structure characterization of hempseed protein isolates. J Food Sci Technol. (2019) 56:3337–46. doi: 10.1007/s13197-019-03815-5, PMID: 31274901 PMC6582010

[43] Zhang S De Leon Rodriguez LM Li FF Brimble MA. Recent developments in the cleavage, functionalization, and conjugation of proteins and peptides at tyrosine residues. Chem Sci. (2023) 14:7782–817. doi: 10.1039/d3sc02543h, PMID: 37502317 PMC10370606

[44] Trumbo P Schlicker S Yates AA Poos M. Food and nutrition Board of the Institute of medicine, the National Academies. Dietary reference intakes for energy, carbohydrate, fiber, fat, fatty acids, cholesterol, protein and amino acids. J Am Diet Assoc. (2002) 102:1621–30. doi: 10.1016/s0002-8223(02)90346-9, PMID: 12449285

[45] He R Girgih AT Malomo SA Ju X Aluko RE. Antioxidant activities of enzymatic rapeseed protein hydrolysates and the membrane ultrafiltration fractions. J Funct Foods. (2013) 5:219–27. doi: 10.1016/j.jff.2012.10.008

[46] Novikov VY Derkach SR Kuchina YA Shironina AY Mukhin VA. Kinetics of enzymatic reactions in the production of fish protein hydrolysates. J Dispers Sci Technol. (2018) 39:1454–61. doi: 10.1080/01932691.2017.1414612

[47] Nguyen HT Bao HND Dang HTT Tómasson T Arason S Gudjónsdóttir M. Protein characteristics and bioactivity of fish protein hydrolysates from Tra catfish (*Pangasius hypophthalmus*) side stream isolates. Food Secur. (2022) 11:4102. doi: 10.3390/foods11244102, PMID: 36553843 PMC9778320

[48] Fadimu GJ Gill H Farahnaky A Truong T. Investigating the impact of ultrasound pretreatment on the physicochemical, structural, and antioxidant properties of Lupin protein hydrolysates. Food Bioprocess Technol. (2021) 14:2004–19. doi: 10.1007/s11947-021-02700-4

[49] Neyra Recky JR Serrano MP Dántola ML Lorente C. Oxidation of tyrosine: antioxidant mechanism of l-DOPA disclosed, free Radic. Biol Med. (2021) 165:360–7. doi: 10.1016/j.freeradbiomed.2021.01.037, PMID: 33516913

[50] Jadhav HB Gogate P Annapure U. Analysing the repercussions of ultrasound on triacylglycerols in food. Food Chem Adv. (2023) 2:100332. doi: 10.1016/j.focha.2023.100332

[51] Ganguly M Debraj D Mazumder N Carpenter J Manickam S Pandit AB. Impact of Ultrasonication on the oxidative stability of oil-in-water Nanoemulsions: investigations into kinetics and strategies to control lipid oxidation. Ind Eng Chem Res. (2021) 63:10212–25. doi: 10.1021/acs.iecr.4c00506, PMID: 40366208

